# Making guidelines computable

**DOI:** 10.1002/gin2.12014

**Published:** 2024-04-04

**Authors:** Brian S. Alper

**Affiliations:** ^1^ Computable Publishing LLC Franklin North Carolina USA; ^2^ Scientific Knowledge Accelerator Foundation Franklin North Carolina USA

Guideline development is easy and efficient. With instant access to all the contributing information—all the relevant evidence, critical appraisal of the evidence by the community, values and preferences of public representatives, judgements by multidisciplinary experts, and re‐usable data where others have developed recommendations for similar decisions—….

Wait. It's 2024, not 2042. Let's try that again.

Guideline development is difficult and resource‐intensive. Even when the decision‐making process works well, there is so much work involved to gather the evidence, assess the certainty of the evidence, determine the relative importance of the outcomes and consider contextual factors. It is sometimes easier if we can adapt from others who have already done it, but their work is not fitting what we need, so we essentially recreate the work, using our development methods anyway.

Some aspects of guideline development are necessarily difficult and should not be oversimplified, but there are many opportunities to reduce the work involved. For example, automating tasks that do not require human cognition, such as identifying direct links to supporting information, can greatly improve work efficiency. To realize this potential, the guideline development content will need to be available in a form the computer can process.

Computers could make guideline development more efficient. They already do, to some degree. We copy and paste instead of retyping when we can. We use autocomplete features to enter data when the machine can guess what we want to express, or dropdown lists when the choices are preset for us. We have come to expect massive increases in efficiency at times, such as rapid responses for targeted searching in large databases. Compare that to literature searching before the Internet.

But the essence of our work—understanding the evidence and judgements sufficiently to select information and use it for informing our decisions—is not grasped by the computer. We may try to apply artificial intelligence (AI) to the challenge and occasionally show a tool helps a step in the process (e.g., highlighting population, intervention, and outcome terms in the text),^1^ but we have yet to create an AI that understands evidence and judgements.

Imagine if we could make the evidence and judgements computable (i.e., machine‐interpretable) so that the computer could create derivative concepts through calculations and logical operations. Searches would be even more efficient. Compare the precision searching for a nearby restaurant when you are travelling to finding evidence for a specific clinical outcome. You can find not only the restaurant's name but also its location, hours of operation and a link to its menu. However, if you find an article that mentions the clinical outcome in the abstract, you still need to obtain the full text, read it to extract the data and make many judgements to determine the certainty of the reported finding. The restaurant data are machine‐interpretable, but the outcome data are not.

Efficiency would be further increased if the knowledge (evidence and judgements) were interoperable, so any computer system could use the output (reuse the work) of any other computer system. Today, a systematic reviewer and guideline developer that use reference management software for citation management, PICO Portal for screening of articles, Robot Reviewer for assistance in risk of bias assessment, the Systematic Review Data Repository (SRDR+) for reporting data extraction, Cochrane RevMan for the meta‐analyses and GRADEpro or MAGICapp for the reporting of summary of findings will need to re‐enter the data for each of these systems. We enjoy extreme efficiency for navigation support due to societal evolution to ubiquitous computable forms of data exchange (see Figure 1) but have yet to achieve this state for evidence and guidelines (see Figure 2).

**Figure 1:**
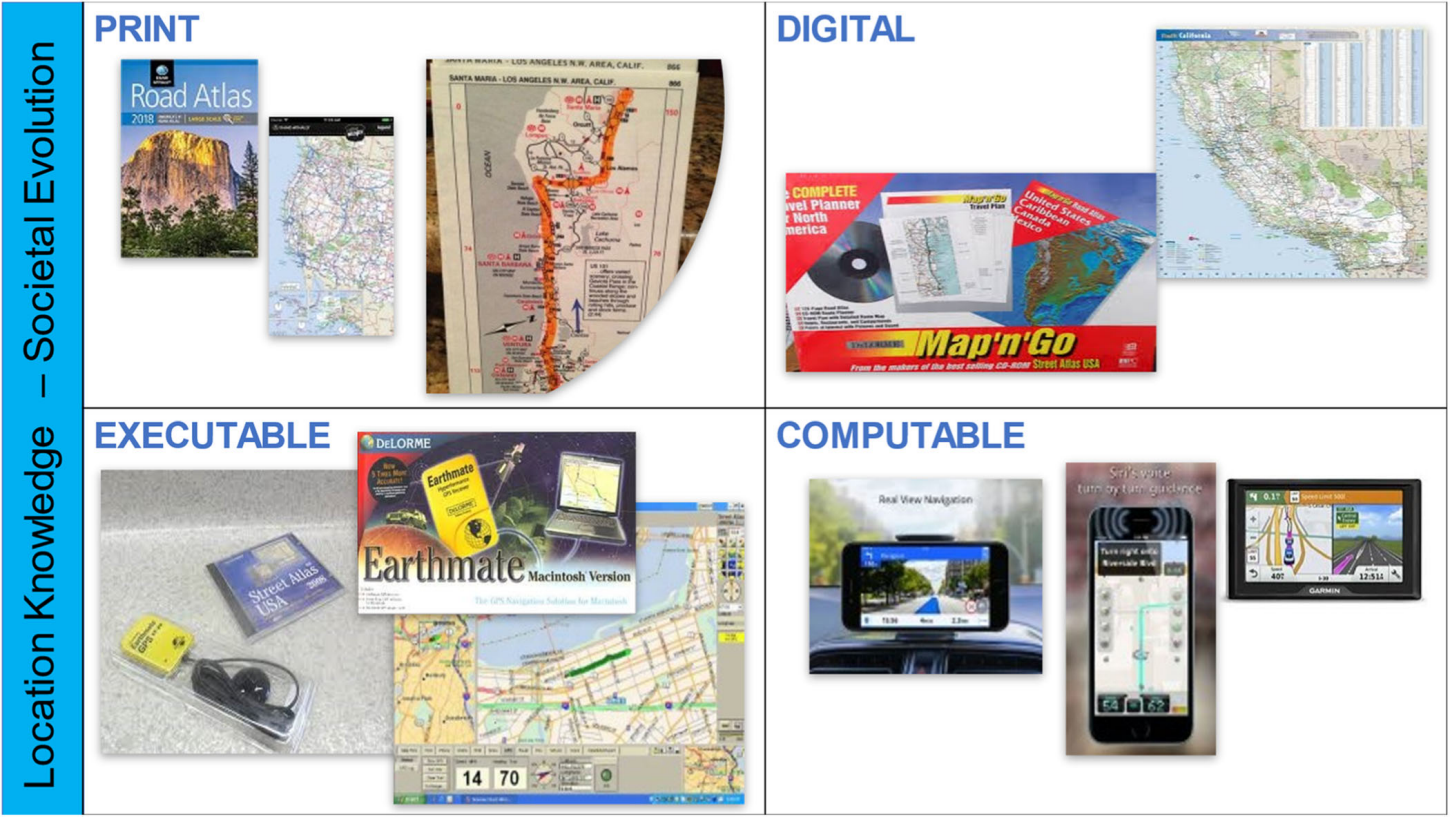
Societal evolution of location data. Presented at Guidelines International Network 2023 conference in Making science computable: guideline‐relevant developments from the Health Evidence Knowledge Accelerator.

**Figure 2:**
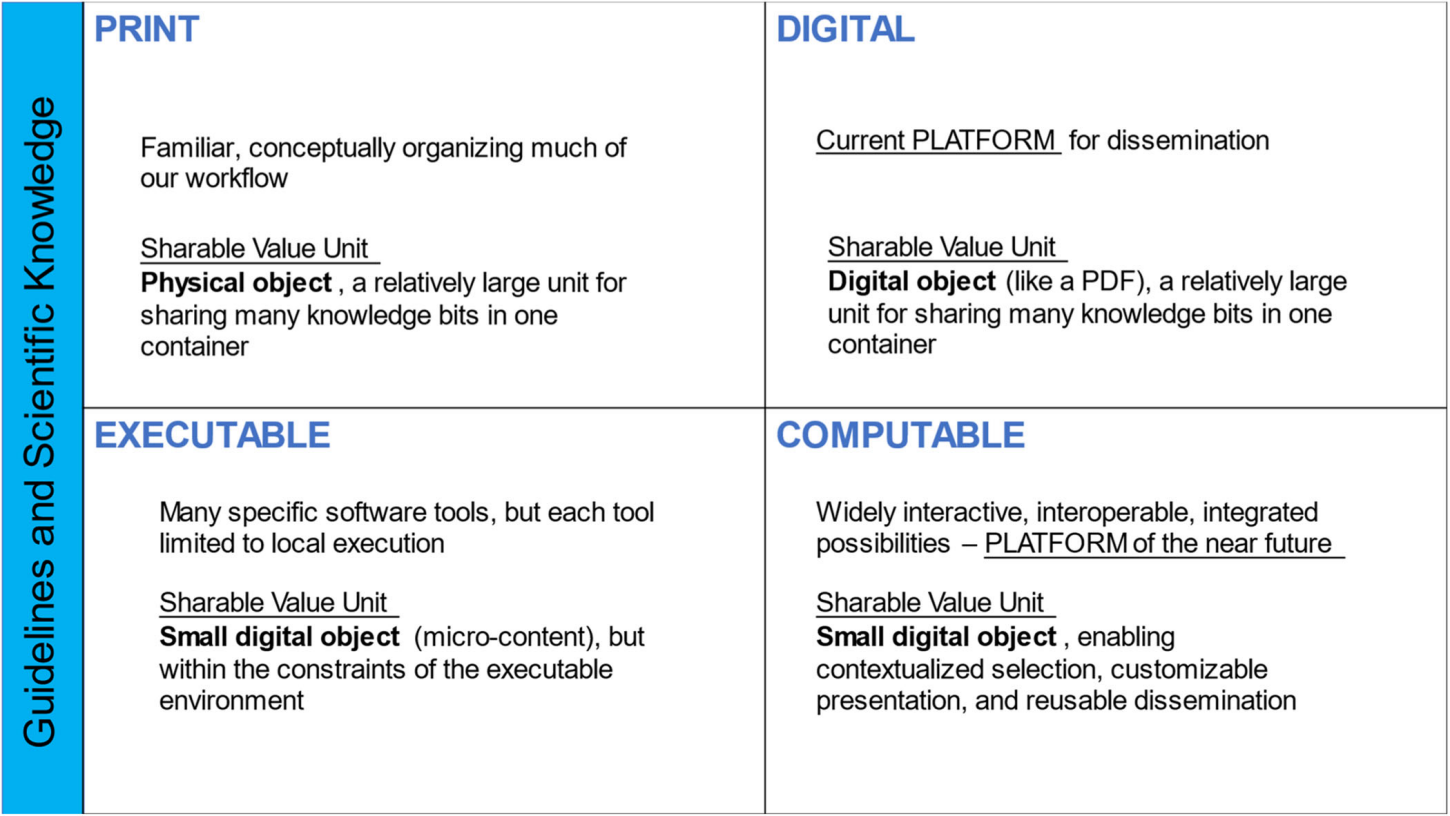
Societal evolution of evidence and guidelines data. Presented at Guidelines International Network 2023 conference in Making science computable: guideline‐relevant developments from the Health Evidence Knowledge Accelerator.

The Guidelines International Network Technology Working Group (GINTech) had a goal in 2017 to achieve interoperable methods to share evidence and guidelines across the ‘Evidence Ecosystem’^2^ but no framework for how to proceed with such a substantial undertaking. In 2018, the present author recognized how a technical standard for health data exchange, Fast Healthcare Interoperability Resources (FHIR), is overcoming the long intractable problem of interoperability for electronic health records.^3^ At its core, FHIR solves the technical problem, by conveying data in small digital packages called Resources, and solves the social agreement challenge, by establishing global consensus through Health Level Seven International (HL7), a standards developing organization.

With this insight, we approached HL7 to extend FHIR to define a standard for data exchange for research results (evidence) and judgements related to certainty of the evidence and making recommendations (evidence‐based guidance). The FHIR Resources for Evidence‐Based Medicine (EBM) Knowledge Assets project (EBMonFHIR) was approved on May 16, 2018, as an HL7 project.^4^

There are now technical standards (standard for data exchange) for how to represent evidence and guidelines in machine‐interpretable, interoperable form.^5^ We defined the structure for an Evidence Resource that precisely represents the variables, the study design, the statistical values, the analytic model and the certainty judgements for a single research finding.^6^ We defined an ArtifactAssessment Resource to represent any comment, rating or classification of a bit of knowledge (also called a knowledge artefact or digital knowledge object).^7^

Profiles enable context‐specific modifications to a Resource, and we defined a RecommendationJustification Profile to use the ArtifactAssessment Resource to represent all the concepts reported in the Evidence‐to‐Decision framework.^8^ We are currently working on an Evidence‐Based Medicine Implementation Guide, which describes 73 profiles of 12 Resources for computable evidence and guidance.^9^

The standard for the form of data exchange (syntactic standard) is only part of the overall solution. We also need a standard for the terminology used (semantic standard), and there is no fit‐for‐purpose standard vocabulary for describing evidence and guidance. We are currently 69% of the way through a multiyear, multidisciplinary effort to define about 600 terms for study design, risk of bias and statistics, manifest as the Scientific Evidence Code System (SEVCO).^10^, ^11^ We also recently started similar efforts with the GRADE Working Group to define terms for certainty of evidence, strength of recommendation and evidence‐to‐decision framework judgements for the GRADE Ontology.^12^

Standards for data exchange (syntactic and semantic) are not enough. System developers need to develop or adapt computer systems to use the standards. Software tools used by researchers, methodologists, clinicians and decision‐makers need to work for the user without the user having to learn FHIR or any of the underlying technical specifications.

Making guidelines computable is compelling to enhance the role of guidelines in the overall ecosystem (see Figure 3), but there are limitations. Many stakeholders need to agree on the precise expectations for knowledge transfer at many different points of data exchange. Neither simple voting nor regulatory mandates can establish the agreements needed to achieve the necessary functionality. Great care must be taken to avoid the illusion of accuracy or correctness that can occur with artificial precision of concepts when ambiguous language is transformed into exacting machine code. The effort to make it easy will not be easy.

**Figure 3:**
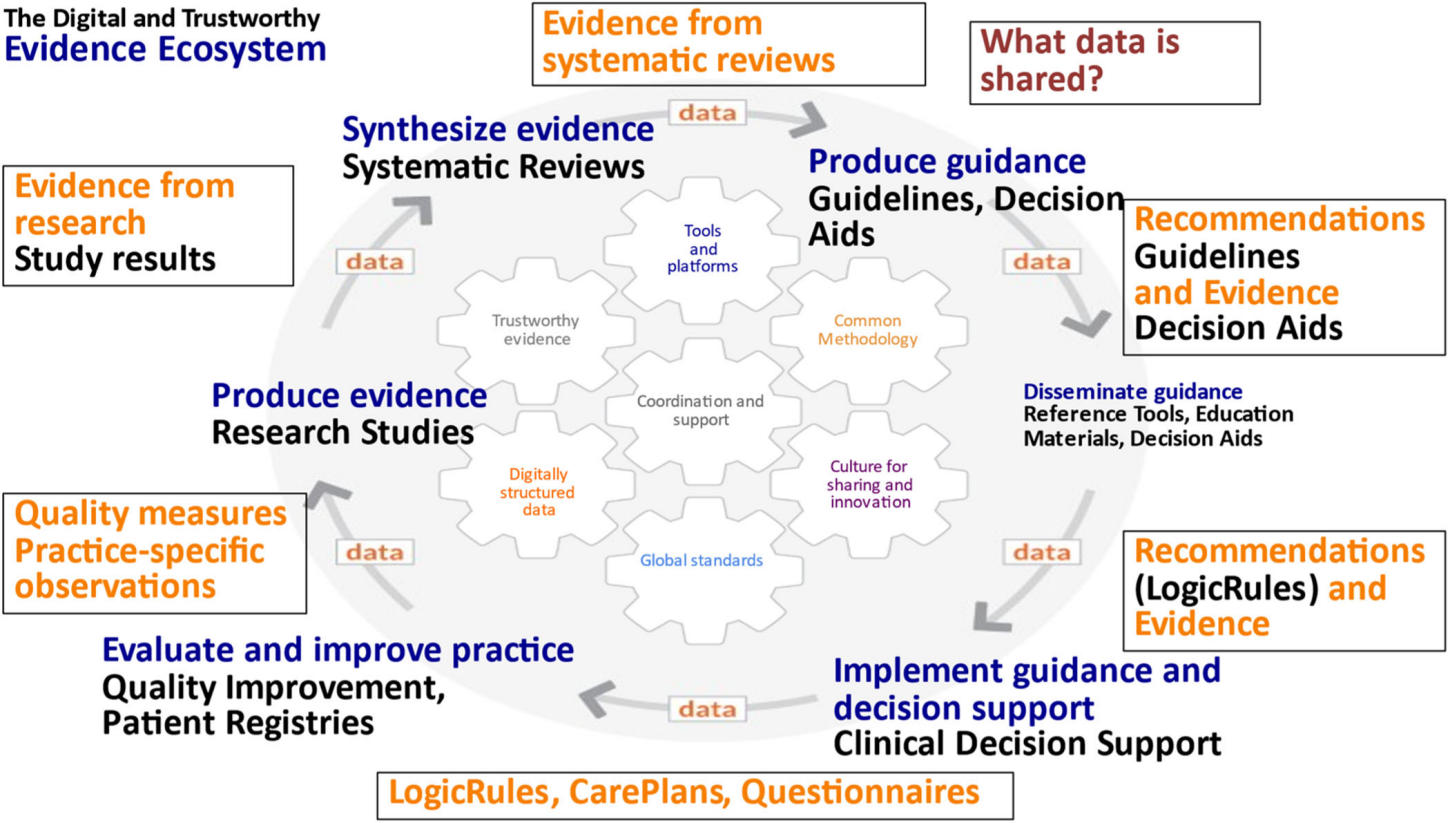
Data sharing across the evidence ecosystem. *Source*: Image adapted from MAGIC Foundation.

Making guidelines computable is an ‘Evidence Ecosystem’‐level community effort. The effort has grown since its inception in 2018, boosted in a large way in 2020 with the formation of a COVID‐19 Knowledge Accelerator.^13^ The effort is now called Health Evidence Knowledge Accelerator (HEvKA) and has 15 working group meetings per week (see Table 1).^14^ There are working groups of interest to researchers, methodologists and software developers. There is no cost or contractual obligation to participate. The standards developed are open and freely available.

**Table 1:** Health Evidence Knowledge Accelerator meeting schedule as of January 2024.

Day/time (Eastern)	Working group	Goals
Monday 8–9 am	Project Management	Managing HEvKA project and FHIR specification change requests
Monday 9–10 am	Setting the Scientific Record on FHIR	Converting data from repositories to FHIR
Monday 10–11 am	CQL Development (a CDS EBMonFHIR sub‐WG)	Using CQL to express executable data
Monday 2–3 pm	Statistic Terminology	SEVCO development for statistic terms
Tuesday 9–10 am	Measuring the Rate of Scientific Knowledge Transfer	Develop methods to measure the rate of scientific knowledge exchange (SKAF Board last Tuesday each month)
Tuesday 2–3 pm	StatisticsOnFHIR (a CDS EBMonFHIR sub‐WG)	Develop FHIR specification and Implementation Guide for statistical data exchange
Tuesday 3–4 pm	Ontology Management	Managing SEVCO and other terminology development efforts
Wednesday 8–9 am	Funding the Ecosystem Infrastructure	Conceptual development and collaboration for sustainability pursuits
Wednesday 9–10 am	Communications (Awareness, Scholarly Publications)	Prepare publications, presentations, websites
Thursday 8–9 am	EBM Implementation Guide (a CDS EBMonFHIR sub‐WG)	Develop a guide for system developers to use FHIR for EBM data exchange
Thursday 9–10 am	Computable EBM Tools Development	Review and advance FEvIR® Platform developments
Friday 9–10 am	Risk of Bias Terminology	SEVCO development for risk of bias terms
Friday 10–11 am	GRADE Ontology	Terminology development for certainty of evidence and judgements for making recommendations
Friday 12–1 pm	Project Management	Managing HEvKA project and FHIR specification change requests

Abbreviations: ‘a CDS EBMonFHIR sub‐WG’, This HEvKA Working Group meeting also serves as an official HL7 Clinical Decision Support Work Group meeting within the EBMonFHIR project; CQL, Clinical Quality Language; EBM, evidence‐based medicine; FHIR, Fast Healthcare Interoperability Resources; GRADE, Grading of Recommendations Assessment, Development, and Evaluation; HEvKA, Health Evidence Knowledge Accelerator; SEVCO, Scientific Evidence Code System; SKAF, Scientific Knowledge Accelerator Foundation; WG, working group.

We have also developed a platform to support data exchange using the FHIR standard for evidence and guidance knowledge. This platform is called the Fast Evidence Interoperability Resources (FEvIR) Platform.^15^ The FEvIR Platform is available for use now but is ‘prerelease’ and not yet scaled for performance handling of millions of records (MEDLINE alone has about 40 million records). Viewing resources on the FEvIR Platform is open without logging in, and there are 26 Viewer Tools supporting human‐friendly views of FHIR Resources. Signing in is free and required to create content (which can then only be edited by the person who created the content). There are 23 Builder Tools enabling the creation of a FHIR Resource without any working knowledge of FHIR. These include a Recommendation Authoring Tool and a Guideline Authoring Tool.^16^, ^17^ There are 16 specialized tools, including Converter Tools which will convert data from MEDLINE, ClinicalTrials.gov, MAGICapp and RIS to FHIR.^18^, ^19^, ^20^, ^21^

Prioritization for tool development on the FEvIR Platform is determined by participation and by resources. We anticipate 2024 priorities to include substantial gain in features for the updating and adapting functions of the recommendation and guideline authoring tools.

With computable guidelines (i.e., specification of guideline content and guideline development content in machine‐interpretable form), guideline developers will be able to spend more of their time making interpretations, judgements and decisions and less of their time re‐entering data, editing for format to fit and refit the system and searching to find specific bits of information.

With all these developments, creating, updating and adapting guidelines will be much easier and much more efficient. And we will get there before 2042.

## CONFLICT OF INTEREST STATEMENT

The author is the owner and CEO of Computable Publishing LLC, a small business providing consulting services and software development and hosting the FEvIR Platform; president of Scientific Knowledge Accelerator Foundation, a nonprofit organization to support virtual scientific knowledge accelerators such as HEvKA; and chair of GIN Tech Working Group, a committee of Guidelines International Network which is collaborating with standards development for data exchange for sharing evidence and guidance in computable form.

## Data Availability

No data set was used for this Commentary. Health Evidence Knowledge Accelerator (HEvKA) projects have data available at https://fevir.net.
